# Identification of a novel variant of LMP-1 of EBV in patients with endemic Burkitt lymphoma in western Kenya

**DOI:** 10.1186/1750-9378-8-34

**Published:** 2013-09-09

**Authors:** Eric M Wohlford, Amolo S Asito, Kiprotich Chelimo, Peter O Sumba, Paul C Baresel, Rebecca A Oot, Ann M Moormann, Rosemary Rochford

**Affiliations:** 1 Center for Global Health and Translational Science, SUNY Upstate Medical University, Syracuse, NY 13210, USA; 2 Center for Global Health Research, Kenya Medical Research Institute, Kisumu, Kenya; 3 Jaramogi Oginga Odinga University of Science and Technology, Bondo, Kenya; 4 Department of Bomedical Science and Technology, Maseno University, Maseno, Kenya; 5 Department of Pediatrics, University of Massachusetts Medical School, Worchester, MA 01655, USA

## Abstract

**Background:**

Epstein Barr virus (EBV) is a gammaherpesvirus that is associated with nasopharyngeal carcinoma (NPC) and endemic Burkitt lymphoma (eBL). EBV carries several latent genes that contribute to oncogenesis including the latent membrane protein 1 (LMP-1), a known oncogene and constitutively active CD40 homolog. Variation in the C terminal region of LMP-1 has been linked to NPC pathogenesis, but little is known regarding LMP-1 variation and eBL.

**Results:**

In the present study, peripheral blood samples were obtained from 38 eBL patients and 22 healthy controls in western Kenya, where the disease is endemic. The LMP-1 C-terminal region from these samples was sequenced and analyzed. The frequency of a 30 base pair deletion of LMP-1 previously linked to NPC was not associated with eBL compared to healthy controls. However a novel LMP-1 variant was identified, called K for Kenya and for the G318K mutation that characterizes it. The K variant LMP-1 was found in 40.5% of eBL sequences and 25.0% of healthy controls. All K variant sequences contained mutations in both of the previously described minimal T cell epitopes in the C terminal end of LMP-1. These mutations occurred in the anchor residue at the C-terminal binding groove of both epitopes, a pocket necessary for MHC loading.

**Conclusions:**

Overall, our results suggest that there is a novel K variant of LMP-1 in Kenya that may be associated with eBL. Further studies are necessary to determine the functional implications of the LMP-1 variant on early events in eBL genesis.

## Background

Epstein Barr virus (EBV) is a well known infectious cofactor involved in the development of several malignancies, including endemic Burkitt lymphoma (eBL) and nasopharyngeal carcinoma (NPC) (reviewed in [1]). Still under question, however, is how EBV functions to drive malignancy. One possibility is that genetic variation in EBV leads to immune evasion of virally infected cells.

**Figure 1:**
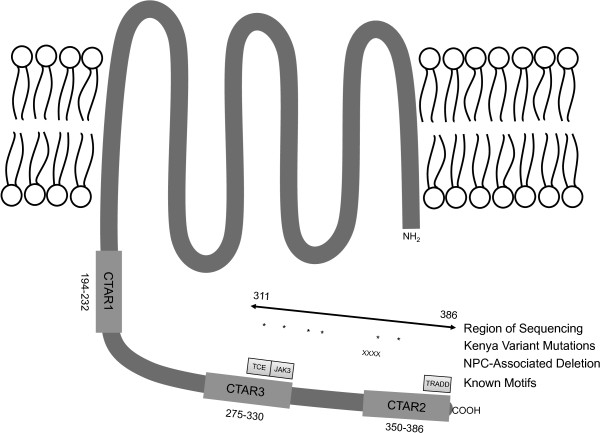
**Diagram of LMP-1 structural and functional motifs.** Cytoplasmic terminal activating regions are labeled CTAR1-3 and labeled with their corresponding amino acid numbers. The region that we sequenced is labeled, along with the positions of amino acid mutations in the K variant sequence, designated with *. The 10 amino acid deletion associated with NPC is labeled with X. The T cell epitope region of CTAR3 is labeled TCE, the JAK3 binding region is labeled JAK3, and the TRADD motif of CTAR2 is labeled TRADD.

EBV encodes a number of genes that contribute to maintaining cell proliferation, blocking apoptosis, and contributing to the malignant phenotype of cancer cells [2-5]. One of the main EBV encoded oncogenes is latent membrane protein-1 (LMP-1) [6]. Latent membrane protein-1 is expressed during primary B cell infection, functioning as a constitutively active CD40 homolog and affecting many cellular proteins including TRADD, JAK3, PI3K, and RIPs [4,7,8]. Overexpression of LMP-1 in EBV-negative cell lines has shown that LMP-1 blocks apoptosis, increases cytokine production, cellular migration and transformation, and decreases cellular adhesion [8,9]. The structure of LMP-1 includes six transmembrane regions starting at the N terminus, with a long cytoplasmic tail containing three C terminal activating regions (CTAR), responsible for activating signaling cascades (Figure 1) [8].

Genetic variation of LMP-1 has been classified using different schemes [10-13]. These schemes were developed from sequences of different geographic areas and cellular origins. Sandvej and colleagues published the first of these classification schemes using a variety of healthy European sequences [12]. In this study Sandvej and colleagues identified 4 variants of LMP-1 sequences in healthy Caucasians and labeled them A, B, C, and D [12]. The most frequent LMP-1 variant observed was variant A (41.2%), followed by variant C (26.5%), variant D (17.6%), variant B (11.8%), and uncharacterized (2.9%) [12]. Previous sequencing studies had been performed using tumor tissue rather than peripheral blood from healthy individuals [14], potentially selecting for certain viral sequences.

Mutations and deletions within the CTARs of LMP-1 have been associated with disease [15-17]. Specifically, a 10 amino acid deletion mutant of LMP-1 as compared to the prototypical B95.8 EBV strain has been associated with NPC cases in Asia, Europe, and North Africa [18-20]. In a retrospective study of EBV-positive lymphoproliferative disorders, the LMP-1 deletion mutant was linked to malignant phenotypes [21]. Deletions in LMP-1 have also been associated with other types of EBV-positive lymphomas [22-24]. One study of children in Turkey with Burkitt lymphoma reported a high frequency of the larger 69 base pair deletion variant of LMP-1, but this study did not compare incidence to healthy controls [25]. A study in Brazil reported that a similar high proportion of Burkitt lymphoma patients and controls harbored deletion variants of LMP-1 [26]. Other studies have examined the association of EBV variants with eBL and produced conflicting results [13,27-30]. Focused studies on EBV variation in eBL patients relative to healthy controls are needed to clarify these divergent observations. To our knowledge, no study has examined the extent of genetic diversity of LMP-1 in an area endemic for BL or in eBL patients.

Genetic variation in LMP-1 has been shown to correlate with differences in T cell immunity [31-33]. Two ways that variant LMP-1 can decrease T cell immunity are through enhancement of regulatory T cells (Tregs) and immune evasion. The role of Tregs in NPC was examined by Pai et al. wherein an NPC-associated LMP-1 variant failed to stimulate T cells as effectively as wildtype LMP-1 in a mixed lymphocyte reaction [33]. The NPC-associated LMP-1 variant led to enhanced IL-10 production by antigen presenting cells, enhancing regulatory T cell function and reducing T cell responses to LMP-1 [33]. LMP-1 is also a target for EBV cytotoxic T lymphocytes (CTL) and has well described T cell epitopes [32,34]. Duraiswami and colleagues showed that there are 6 LMP-1 peptide sequences that stimulate LMP-1 specific T cells to produce IFN-γ. Each of these regions was broken down into the minimal peptide sequences that were T cell epitopes. One of the T cell epitope regions within LMP-1 falls within CTAR3 [34], an area with known sequence variation [11,12,35]. A sequencing study of LMP-1 T cell epitopes from NPC patients showed no association with disease, however it has not been shown whether LMP-1 variation within the T cell epitope region is associated with immune evasion in eBL [34]. While LMP-1 is not expressed in eBL, T cell control of EBV during primary infection of B cells may be impaired by different LMP-1 variants.

The current study sought to answer several outstanding questions. First, what is the diversity of LMP-1 sequence variation in an area endemic for eBL? Second, are certain LMP-1 genotypes associated with eBL compared to healthy controls? Finally, what does LMP-1 variation suggest about EBV pathogenesis? To answer these questions the C terminus of LMP-1 was sequenced from eBL patients and healthy controls from an eBL endemic area of western Kenya. A novel LMP-1 variant was observed in the Kenyan population, was highly prevalent in eBL patients, and carried mutations in the C terminal amino acids of both minimal T cell epitopes found in the portion of LMP-1 studied. These results may have implications for EBV-mediated immune evasion in the early events of Burkitt lymphomagenesis.

## Results

### Study populations

Endemic Burkitt lymphoma patients and healthy controls were selected based on their availability from our previously reported case control study [36]. In this study only 13% of eBL patients were parasitemic by blood smear at admission, although nearly all resided in a malaria holoendemic area [37]. Also 28% of parents reported giving their child antimalarial treatment in the two weeks prior to presentation (Moormann, unpublished observation). Therefore point prevalence malaria status for eBL patients at presentation to this tertiary care hospital is not an accurate indicator of recent malaria. We have previously reported that 68% of this group of healthy controls were malaria positive at sampling [36]. Additional controls (C17-C24) were included from a nearby area of western Kenya [38], and of these 57% were PCR positive for malaria. Although acute malaria increases EBV load and possibly detectability [39], we were able to amplify EBV DNA from all eBL patients and healthy controls sampled, suggesting a low rate of detection bias of EBV. After sequencing it was pathologically determined that two eBL patients had tumors other than eBL (BL16 and BL39), and their sequencing data were excluded from the analysis but can be found in Additional file 1: Table S1. The mean age of eBL patients was 90 months and for healthy controls was 54 months. For eBL patients 56.8% were male and for healthy controls, 40.9% were male. A summary of demographic data on the study populations is shown in Table 1.

**Table 1:** Demographic characteristics of study participants

**Group**s	**Number enrolled**	**Coinfections**	Excluded (n)*	**Analyzed (n)**	**Mean age (mo)**	**Age range (mo)**	**Sex (% male)**
eBL	38	1	2	37	90	36-161	56.8
Control	22	2	0	24	54	4-144	40.9

n – number of samples.

mo – months.

*Samples were excluded if pathological diagnosis was not Burkitt lymphoma.

### Coinfection with multiple EBV variants

**Figure 2:**
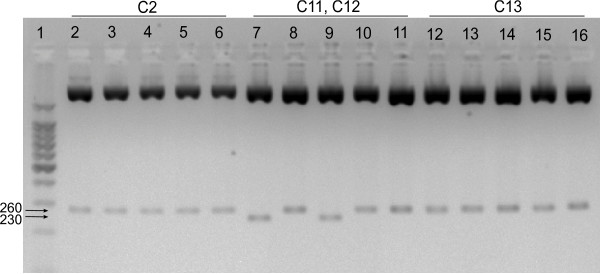
**Gel electrophoresis image of plasmid digestion from three study participants.** Lane 1 is the 100 base pair ladder, 500 bp has increased intensity. Lanes 2-6 are five clones from participant C2, lanes 7-11 are from participant C11-C12, and lanes 12-16 are from participant C13. The full-length product (~260 bp) is visible in all five clones from C2 and C13. The 30 base pair deletion mutant (~230 bp) is visible in two clones (lanes 7 and 9, C11) of participant C11-C12.

Coinfection with different EBV LMP-1 deletion variants was determined by difference in the product size among clones. One eBL patient and two healthy controls had two discernible variants in LMP-1 size as determined by the size of the cloned PCR product when analyzed by gel electrophoresis (Figure 2). Both of the variants for the three study participants were sequenced and pooled with the results of the remaining sequences for analysis, resulting in 39 eBL sequences and 24 healthy control sequences.

### Diversity of LMP-1 sequence variants

**Figure 3:**
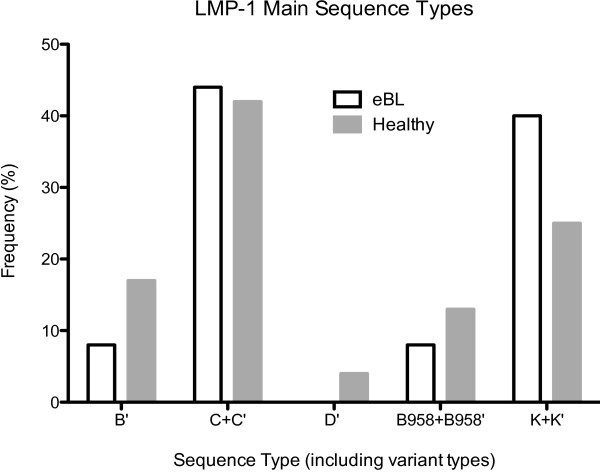
**Frequency of all LMP-1 variants between healthy control eBL patient samples.** Bars represent the frequency of each LMP-1 type, including amino acid variants, e.g. K+K’. White bars represent eBL sequences and gray bars represent healthy controls.

The T cell epitope region of CTAR3 through the 30 base pair deletion region to the 3′ end of the LMP-1 gene that was sequenced is shown in Figure 1. Isolates were then categorized into the scheme defined by Sandvej and colleagues and also compared with the prototypic B95.8 strain of EBV [15,35]. Because Sandvej et al. sequenced LMP-1 from many healthy Europeans [12], and compared the sequences to lymphoma patients [35], this classification scheme was chosen for the present study. In the present study of the C terminus of LMP-1, in contrast to Sandvej et al., variant A was not observed, while B, C, D, and B95.8 EBV LMP-1 variants were observed. Table 2 represents the full array of mutations observed in this study population, and the frequency of each variant in healthy control and eBL samples is shown in Figure 3. The only variant sequence represented exactly as described by Sandvej was the C variant, which was present in 15 (40.5%) eBL sequences and 7 (29.2%) control sequences (p=0.42, OR 1.65, 95% CI 0.55-4.97). However other variants could be characterized as similar to C type, differing only by single amino acid substitutions. These variants were denoted C’ and when combined with true C variant totaled 17 (45.9%) eBL samples and 10 (41.7%) healthy controls (p=0.80, OR 1.19, 95% CI 0.42-3.36). Thus no difference in the frequency of C variant was observed between eBL and healthy control sequences.

**Table 2:** Location of all amino acid mutations present in this study

**Number of Samples**	**LMP-1 Type**	**T cell epitope**																					
		**CTAR 3**		**JAK binding site**													**CTAR 2**						
		**Amino acid position**																					
		**313**	**318**	**321**	**322**	**328**	**331**	**334**	**338**	**343**	**344**	**345**	**346**	**347**	**348**	**349**	**350**	**351**	**352**	**354**	**356**	**366**	**372**
	**B95.8 RefSeq**^**#**^	**S**	**G**	**P**	**Q**	**E**	**G**	**Q**	**L**	**G**	**G**	**G**	**H**	**S**	**H**	**D**	**S**	**G**	**H**	**G**	**D**	**S**	**D**
1	B'		N		E			R	S										R			T	
3	B'				E			R	S										R			T	
2	B'				E			R	S													T	
1	B95.8																						
1	B95.8'																				N		
3	B95.8'																					T	
1	B95.8'																		N			T	
22	C				N			R	S		*	*	*	*	*	*	*	*	*			T	
1	C'				N	G		R	S		*	*	*	*	*	*	*	*	*			T	
2	C'				N			R	S		*	*	*	*	*	*	*	*	*			T	H
1	C'				N			R	S		*	*	*	*	*	*	*	*	*	S		T	G
1	C'			S	N			R	S		*	*	*	*	*	*	*	*	*			T	
10	K'		K		E			R	S													T	
11	K		K		E			R	S										R			T	
1	D'	P			T		Q		P													T	

Sequences are grouped according to the scheme devised by Sandvej et al. [12], with amino acid mutations labeled at sites along LMP-1 protein sequence. Novel K variant amino acid changes are also labeled.

^#^ Stands for B95.8 reference sequence. * Stands for amino acid deletion.

Variants of several other previously described LMP-1 isolates were observed, including B, D, and B95.8. There were no prototypical B strains, but 2 (7.7%) eBL sequences and 4 (16.7%) healthy control sequences differed by only one to two amino acids from the prototypical B strain. There was no significant difference in the proportion of B variant sequences between these two groups (p=0.20, OR 0.29, 95% CI 0.05-1.70). A single D variant strain, which differed from the prototypical D strain by two amino acids, was present in one healthy control sequence and no eBL sequences (p=0.39, OR 0.21, 95% CI 0.01-5.35). One prototypical B95.8 sequence occurred in an eBL patient. There were five B95.8 amino acid variants, 3 (12.5%) from healthy control sequences, and 2 (5.4%) from eBL sequences (p=0.37, OR 0.40, 95% CI 0.06-2.59). When these were analyzed together with the prototypical B95.8 sequence, no statistically significant difference in frequency of B95.8 variant was observed between eBL sequences and healthy controls (p=0.67, OR 0.62, 95% CI 0.11-3.35).

**Figure 4:**
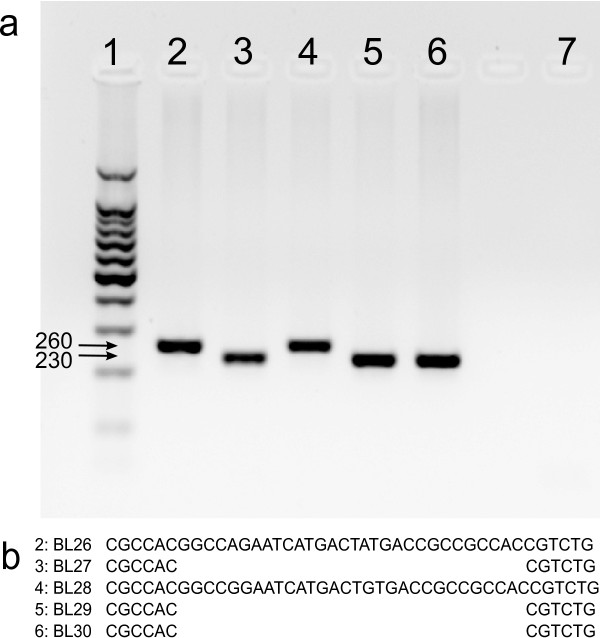
**Confirmation of agreement between gel electrophoresis and sequencing result.** Patient BL26 and BL28 contained the full-length LMP-1 product, while BL27, BL29, and BL30 contained deletion variants by both electrophoresis and sequencing. Part **a** is a sample gel electrophoresis image from a PCR amplification of five eBL patient LMP-1 sequences. Lane 1 is a 100 base pair ladder, with 500 base pairs highlighted. Lane 2 is from patient BL26, lane 3 is from BL27, lane 4 is from BL28, lane 5 is from BL29, lane 6 is from BL30. Lane 7 is a no template PCR control. Part **b** represents the sequence traces of the corresponding eBL patient samples flanking the 30 base pair deletion.

Presence of the 30 base pair deletion LMP-1 mutant detected by gel electrophoresis or by sequencing was compared and 100% concordance was observed between electrophoresis and sequencing studies in detecting the LMP-1 deletion (Figure 4, other data not shown). Next the frequency of the deletion mutant was compared between eBL cases and healthy controls. The 30 base pair deletion mutant was present in 17 (45.9%) eBL sequences and 10 (41.7%) healthy controls (p=0.80, OR 1.19, 95% CI 0.42-3.36).

No mutations were observed in the TRADD/RIP binding sequence of CTAR2, which occurs from amino acids 379-385 of LMP-1. Of the 63 sequence reads, 55 produced clean traces through the end of the LMP1 coding sequence. The other 8 sequences were amplified with primers that did not include the last 8 amino acids of LMP-1, and this portion has been excluded from their analysis. However in all 55 traces, the TRADD/RIP binding motif at the C terminal end of CTAR2 was 100% conserved in all samples.

### Novel K variant of LMP-1

A previously uncharacterized LMP-1 variant was observed in both eBL patients and healthy controls. This variant always differed from the B95.8 sequence at 5 amino acids: G318K, Q322E, Q334R, L338S, and S366T; and was frequently found with H352R (52.4%). We have named the novel variant K for Kenya and for the novel lysine substitution at amino acid 318. The prototypical K variant was found in 9 (24.3%) eBL sequences and 2 (8.3%) healthy controls (p=0.18, OR 3.54, 95% CI 0.69-18.07). The atypical K variant containing H352R was found in 6 (16.2%) eBL sequences and 4 (16.7%) healthy controls (p=1.00, OR 0.97, 95% CI 0.24-3.87). When the prototypical K variant was combined with atypical K variant sequences for analysis there was no difference in frequency between eBL sequences and controls (p=0.27, OR 2.05, 95% CI 0.66-6.36).

### LMP-1 T cell epitope variants

**Figure 5:**
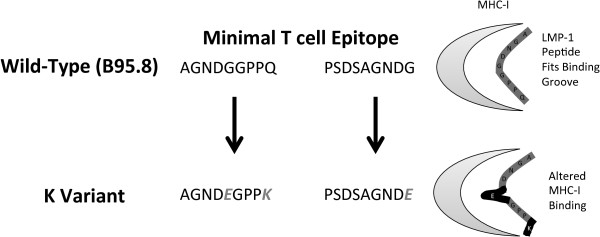
**Diagram of minimal T cell epitopes in CTAR3 of wild-type EBV and mutations in K variant LMP-1.** Highlighted are how known peptides fit into MHC-I and possible effects of mutations on MHC processing.

Duraiswami and colleagues showed that only specific LMP-1 epitopes are able to elicit interferon-γ production from T cells [34]. One of these epitopes occurs in CTAR3, from amino acids 307 to 323. Within this region it was determined that there were two minimal sequences of 9 amino acids necessary for recognition by EBV-specific T cells. The minimal T cell epitope sequences within CTAR3 were AGNDGGPPQ and PSDSAGNDG. When the K sequence was mapped onto these epitopes, it was found that the K variant was mutated at the C terminal amino acid of both minimal T cell epitopes, creating sequences AGND**E**GPP**K** and PSDSAGND**E**. A diagram of the possible effects of these mutations on MHC-I loading is shown in Figure 5. The G318K mutation was highly linked to the Q322E mutation, such that all 22 sequences observed containing G318K also contained Q322E.

An amino acid mutation at Q322 in the C terminal of the T cell epitope was detected in 55 of 61 samples analyzed. While all K variant sequences contained two amino acid mutations in the T cell epitope region of CTAR3, all but two other sequences with mutations in this region harbored mutations only in Q322. Of the two sequences with multiple T cell epitope mutations, one was an alternate C variant sequence (BL36), with mutations in both terminal amino acids, to AGNDGGP**SN**. The other was a B variant sequence (C2), and contained the sequence AGND**N**GPP**E**.

## Discussion

The main goals of this study were to determine the genetic variation of the C terminus of LMP-1 in children residing in western Kenya, whether variation was linked to eBL versus healthy controls, and what LMP-1 variation suggests about EBV biology. To address the first goal of our study, the LMP-1 sequences obtained from Kenyan study participants were compared to previously reported sequences from healthy Caucasians [12]. The major LMP-1 sequences observed in the Kenyan population were the C variant and a previously unreported K variant sequence. We are unaware of any previous studies describing the characteristic G318K mutation of the K variant sequence. Other LMP-1 variants observed included the B, D, and B95.8. No A variant sequences were observed among this population from western Kenya, in contrast to the high prevalence observed in the European population [12]. This general pattern of EBV variants could suggest historical movement of EBV among populations [11]. For example, the A variant virus in the European population may have arisen independently of mutation in the African setting. Further studies using larger regions of the EBV genome and sequences from diverse geographical regions are necessary to validate these observations across the global population.

The second aim of this study was to determine if certain LMP-1 genotypes were associated with eBL as compared to healthy controls. None of the previously characterized LMP-1 variants observed were associated with eBL, including B, C, D, and B95.8. The novel K variant LMP-1 was found in 40.5% of eBL sequences and 25.0% of healthy controls (p=0.27). Larger sample sizes are needed to confirm whether K variant LMP-1 is associated with eBL in Kenya. Still undetermined is whether the K variant sequence is associated with eBL in other areas endemic for Burkitt lymphoma, which would support an immune evasive phenotype of K variant LMP-1, or if it arose independently in the Kenyan population. The selection of EBV genetic variants in cancer agrees with previous work suggesting that EBV-associated Hodgkin’s disease selects for certain LMP-1 variants, which differ from the distribution of variants in the general population [35]. Similarly in eBL, previous work on EBNA-1 has suggested that certain EBNA-1 variants are more oncogenic than others [27]. Although some research has suggested the selection of specific EBNA-1 genetic variants in lymphomas, other work has suggested that specific EBNA-1 variants are associated only with geographic areas and not with eBL [28].

T cell control of EBV is critical for the development of protective immunity [40]. It was recently confirmed in a mouse model that T cell control of LMP-1 is necessary for inhibiting lymphomagenesis [41]. It has also been determined that only specific LMP-1 epitopes generate interferon-γ responses from T cells [34]. The possible link to T cell immune evasion in K variant LMP-1 derives from the mutated anchor residues in the C terminal binding groove of both of the two known minimal T cell recognition sequences of CTAR3 in the K variant. In addition to their specific location within the anchor position, these mutations resulted in changes in the polarity of the amino acid. The first mutation was from the small and uncharged glycine at position 318 to larger and positively charged lysine. The second mutation at amino acid 322 was from uncharged glutamine to negatively charged glutamic acid. Mutations in the C terminal binding groove affect the ability of peptides to be loaded onto appropriate MHC class I molecules [42,43], so these mutations may play an important role in MHC loading, decreasing the ability of LMP-1 derived peptides to be presented at the cell surface. Our study did not evaluate the MHC specificity of these variants, but the Kenyan population has very high MHC heterogeneity [44], and it is possible that people with certain MHC variants are unable to present these novel LMP-1 peptides. Functional studies are necessary to characterize the MHC specificity of the novel LMP-1 variants identified in this study.

Given the immune evasion hypothesis it is interesting that we did not observe a difference in the frequency of K type LMP-1 between eBL patients and controls. There are multiple possible explanations for this. One possiblity is that the sample size of the current study was too small to detect a difference between these populations. Sampling a larger population was unfortunately not possible for this study. Another possibility is that LMP-1 variants of eBL patients and controls differ in critical T cell epitopes outside of the region sequenced here. It is known that LMP-1 T cell epitopes exist outside of CTAR2 and that amino acid variation leads to functional consequences [34], so this remains a possibility that should be examined by future studies. Another possibility is that K type LMP-1 in healthy individuals clusters spatially with high-risk eBL clusters [37,45]. Spatial data were not recorded in the current study, possibly altering the frequency of K type LMP-1 that would be observed in high versus low risk healthy controls. We believe that future studies including the entire coding region of LMP-1 with larger sample sizes will help resolve this apparent discrepancy.

A major limitation of this study was that LMP-1 was sequenced from DNA extracted from peripheral blood lymphocytes rather than eBL tumor tissue. We were unable to obtain biopsy tissue for these studies. However previous work showed that EBV isolated from eBL biopsy samples contained the same EBNA-1 sequence as EBV obtained from peripheral blood of the same individual, indicating that tumor and peripheral blood EBV isolates were genetically identical [28].

## Conclusions

The C-terminus of LMP-1 was sequenced from peripheral blood of eBL patients and healthy controls in western Kenya. The Kenyan population demonstrated an altered distribution of LMP-1 variants compared to previous studies in Europe. A previously undocumented LMP-1 variant was also observed, called K for Kenya and its novel lysine (K) substitution. The K variant LMP-1 is characterized by amino acid mutations in the C terminal anchor residues of both minimal T cell epitopes of LMP-1 CTAR-3, which may lead to functional differences in MHC loading. The K variant was found at increased frequency in eBL patients compared to healthy controls. Since this variant has not been described in eBL samples previously, larger patient populations will need to be studied to confirm the linkage between K variant and eBL development. Future studies are also needed to confirm the functional role of K variant mutations on MHC loading and T cell immune evasion.

## Methods

### Samples

Endemic BL patients were enrolled when presenting to the New Nyanza Provincial General Hospital in Kisumu, Kenya and healthy controls were enrolled from a nearby malaria holoendemic area as previously described, [46]. Additional controls (C17-C24) were included from a subset of samples of a separate study of healthy children living in a nearby area of Kisumu, Kenya [38]. After obtaining informed consent, approximately five milliliters of peripheral blood was drawn from children with eBL and healthy controls. Whole blood was frozen at -80°C until use. From these frozen samples, 38 eBL patients and 22 healthy controls were randomly selected for sequencing. After beginning the study it was pathologically determined that 2 eBL patients (BL16 and BL39) had non-eBL tumors and their sequencing data were excluded from analysis.

### Ethical approval

Ethical approval was obtained from the Institutional Review Boards at The State University of New York Upstate Medical University (Rochford), The University of Massachusetts Medical School (Moormann), and the Ethical Review Committee at the Kenya Medical Research Institute, Nairobi, Kenya. Parents of minor study participants provided individual, written informed consent in accordance with the Declaration of Helsinki.

### DNA extraction

DNA was extracted from whole blood using the QIAamp DNA Mini Kit (Qiagen, Germantown, MD, USA) according to the manufacturer’s instructions.

### PCR amplification

The LMP-1 segment spanning the 3′ T cell epitope and JAK binding site of CTAR3 as well as CTAR2 was amplified using the following primers of sequence NC_007605.1: 5′-GCGACTCTGCTGGAAATGAT-3′ (167912-31) and 5′-GACATGGTAATGCCTAGAAG-3′ (167672-91). For control samples C17 through C24, primers were 5′-CCGTGGGGGTCGTCATCATC-3′ (167730-49) and 5′-CTCCCGCACCCTCAACAAGC-3′ (168262-43). Primers were acquired from Integrated DNA Technologies (Coralville, IA, USA). Each PCR reaction mixture contained 2.5 μl 10× PCR Buffer, 2.5 μl dNTP mixture, 1.25 μl RedTaq Polymerase (Sigma, Saint Louis, MO, USA), 2.5 μl LMP-1 forward and reverse primers at 3 uM, and 11.75 μl molecular grade water (Mediatech, Herndon, VA, USA). The amplification procedure consisted of a 95°C denaturation step for 10 minutes, followed by 50 cycles of 95°C for 30 seconds, 58°C for 30 seconds, and 72°C for 45 seconds. Reactions were carried out in an iCycler thermocycler (BioRad, Hercules, CA, USA). Positive control DNA was amplified from the EBV positive cell line B95.8. PCR product size was confirmed by gel electrophoresis using a 2% AquaPor agarose (National Diagnostics, Atlanta, GA, USA) gel containing 5% ethidium bromide (Sigma, Saint Louis, MO, USA) at 10mg/ml.

### Cloning

After confirming the appropriate product length, PCR products were cloned using the TOPO TA pCR 2.1 cloning kit with TOP10 chemically competent *Escherichia coli* according to the manufacturer’s instructions (Invitrogen, Carlsbad, CA, USA). Five clones per sample were selected and run on an agarose gel to visualize the presence of the LMP-1 product and the size of the amplicon.

Plasmid DNA was purified from *E. coli* using a Qiagen Plasmid Purification Mini Kit (Germantown, MD, USA) according to the manufacturer’s instructions and eluted in HPLC grade water. To confirm the presence of the LMP-1 insert, plasmid DNA was digested with EcoR1 (New England Biolabs, Ipswitch, MA, USA) according to the manufacturer’s instructions. A total of 5 clones per sample were digested. Digestion products were run on a 2% agarose gel as described above to confirm the presence of LMP-1 insert DNA.

### Sequence analysis

Plasmids containing cloned LMP-1 PCR products were sent to Genewiz (South Plainfield, NJ, USA) for sequencing using M13R universal primers. Sequences were aligned using Unipro UGENE software (Novosibirsk, Russia).

### Statistical analysis

Fisher’s exact test with odds ratios (OR), and 95% confidence intervals (95% CI) in GraphPad Prism, version 5.0b (La Jolla, CA, USA) were used to compare the frequency of LMP-1 variants between eBL patients and healthy controls.

## Competing interests

The authors declare that they have no competing interests.

## Authors’ contributions

RR and AM designed the field study. AA, KC, and PS oversaw and carried out field collections and blood preparation. EW, PB, and RO conducted molecular genetic studies and sequence alignments. EW conducted statistical analyses and drafted the manuscript. All authors read and approved the final manuscript.

## Supplementary Material

Additional file 1: Table S1 Amino acid sequences of patients excluded from study with non-BL tumors. Click here for file

## References

[B1] Kutok JL Wang F Spectrum of Epstein-Barr virus-associated diseases Annu Rev Pathol 2006 1 375 404 10.1146/annurev.pathol.1.110304.100209 18039120

[B2] Vereide D Sugden B Proof for EBV’s sustaining role in Burkitt’s lymphomas Semin Cancer Biol 2009 19 389 393 10.1016/j.semcancer.2009.07.006 19628040 PMC2789873

[B3] Shimizu N Tanabe-Tochikura A Kuroiwa Y Takada K Isolation of Epstein-Barr virus (EBV)-negative cell clones from the EBV-positive Burkitt’s lymphoma (BL) line Akata: malignant phenotypes of BL cells are dependent on EBV J Virol 1994 68 6069 6073 8057484 10.1128/jvi.68.9.6069-6073.1994 PMC237015

[B4] Zhang XN Huang PC Cell survival and death program modulated by LMP1: implication in antitumor immunity Chinese journal of cancer 2009 28 831 837 10.5732/cjc.009.10077 19664329

[B5] Kelly GL Long HM Stylianou J Thomas WA Leese A Bell AI Bornkamm GW Mautner J Rickinson AB Rowe M An Epstein-Barr virus anti-apoptotic protein constitutively expressed in transformed cells and implicated in burkitt lymphomagenesis: the Wp/BHRF1 link PLoS Pathog 2009 5 e1000341 10.1371/journal.ppat.1000341 19283066 PMC2652661

[B6] Klein E Teramoto N Gogolák P Nagy N Björkholm M LMP-1, the Epstein-Barr virus-encoded oncogene with a B cell activating mechanism similar to CD40 Immunol Lett 1999 68 147 154 10.1016/S0165-2478(99)00044-9 10397170

[B7] Zhang Z Zhang Q Yu Y Ouyang Y He Z Construction and function analysis of the Epstein-Barr virus-encoded latent membrane protein-1 of CTAR3 region Wei sheng wu xue bao = Acta microbiologica Sinica 2008 48 1308 1313 19160809

[B8] Li HP Chang YS Epstein-Barr virus latent membrane protein 1: structure and functions J Biomed Sci 2003 10 490 504 10.1007/BF02256110 12928589

[B9] Bentz GL Whitehurst CB Pagano JS Epstein-Barr Virus Latent Membrane Protein 1 (LMP1) C-Terminal-Activating Region 3 Contributes to LMP1-Mediated Cellular Migration via Its Interaction with Ubc9 ▿ J Virol 2011 85 10144 10153 10.1128/JVI.05035-11 21795333 PMC3196420

[B10] Edwards RH Seillier-Moiseiwitsch F Raab-Traub N Signature amino acid changes in latent membrane protein 1 distinguish Epstein-Barr virus strains Virology 1999 261 79 95 10.1006/viro.1999.9855 10441557

[B11] Walling DM Shebib N Weaver SC Nichols CM Flaitz CM Webster-Cyriaque J The molecular epidemiology and evolution of Epstein-Barr Virus: sequence variation and genetic recombination in the latent membrane protein-1 gene J Infect Dis 1999 179 763 774 10.1086/314672 10068570

[B12] Sandvej K Gratama JW Munch M Zhou XG Bolhuis RL Andresen BS Gregersen N Hamilton-Dutoit S Sequence analysis of the Epstein-Barr virus (EBV) latent membrane protein-1 gene and promoter region: identification of four variants among wild-type EBV isolates Blood 1997 90 323 330 9207468

[B13] Kanai K Satoh Y Saiki Y Ohtani H Sairenji T Difference of Epstein-Barr virus isolates from Japanese patients and African Burkitt’s lymphoma cell lines based on the sequence of latent membrane protein 1 Virus genes 2007 34 55 61 10.1007/s11262-006-0010-y 16917741

[B14] Miller WE Edwards RH Walling DM Raab-Traub N Sequence variation in the Epstein-Barr virus latent membrane protein 1 J Gen Virol 1994 75 Pt 10 2729 2740 7931159 10.1099/0022-1317-75-10-2729

[B15] Fielding CA Sandvej K Mehl A Brennan P Jones M Rowe M Epstein-Barr virus LMP-1 natural sequence variants differ in their potential to activate cellular signaling pathways J Virol 2001 75 9129 9141 10.1128/JVI.75.19.9129-9141.2001 11533177 PMC114482

[B16] Sung NS Edwards RH Seillier-Moiseiwitsch F Perkins AG Zeng Y Raab-Traub N Epstein-Barr virus strain variation in nasopharyngeal carcinoma from the endemic and non-endemic regions of China International journal of cancerJournal international du cancer 1998 76 207 215 10.1002/(SICI)1097-0215(19980413)76:2<207::AID-IJC7>3.0.CO;2-0 9537582

[B17] Edwards RH Sitki-Green D Moore DT Raab-Traub N Potential selection of LMP1 variants in nasopharyngeal carcinoma J Virol 2004 78 868 881 10.1128/JVI.78.2.868-881.2004 14694118 PMC368819

[B18] See HS Yap YY Yip WK Seow HF Epstein-Barr virus latent membrane protein-1 (LMP-1) 30-bp deletion and Xho I-loss is associated with type III nasopharyngeal carcinoma in Malaysia World J Surg Oncol 2008 6 18 10.1186/1477-7819-6-18 18275617 PMC2265716

[B19] Dardari R Khyatti M Cordeiro P Odda M ElGueddari B Hassar M Menezes J High frequency of latent membrane protein-1 30-bp deletion variant with specific single mutations in Epstein-Barr virus-associated nasopharyngeal carcinoma in Moroccan patients Int J Cancer 2006 118 1977 1983 10.1002/ijc.21595 16287066

[B20] Plaza G Santón A Vidal AM Bellas C Latent membrane protein-1 oncogene deletions in nasopharyngeal carcinoma in Caucasian patients Acta Otolaryngol 2003 123 664 668 12875592

[B21] Kingma DW Weiss WB Jaffe ES Kumar S Frekko K Raffeld M Epstein-Barr virus latent membrane protein-1 oncogene deletions: correlations with malignancy in Epstein-Barr virus–associated lymphoproliferative disorders and malignant lymphomas Blood 1996 88 242 251 8704180

[B22] Tai Y-C Kim L-H Peh S-C High frequency of EBV association and 30-bp deletion in the LMP-1 gene in CD56 lymphomas of the upper aerodigestive tract Pathol Int 2004 54 158 166 10.1111/j.1440-1827.2003.01602.x 14989738

[B23] Sugita Y Terasaki M Niino D Ohshima K Fumiko A Shigemori M Sato Y Asano N Epstein-Barr virus-associated primary central nervous system lymphomas in immunocompetent elderly patients: analysis for latent membrane protein-1 oncogene deletion and EBNA-2 strain typing J Neurooncol 2010 100 271 279 10.1007/s11060-010-0191-z 20455004

[B24] Pongpruttipan T Kummalue T Bedavanija A Khuhapinant A Ohshima K Arakawa F Niino D Sukpanichnant S Aberrant antigenic expression in extranodal NK/T-cell lymphoma: a multi-parameter study from Thailand Diagn Pathol 2011 6 79 10.1186/1746-1596-6-79 21867533 PMC3170575

[B25] Tacyildiz N Cavdar AO Ertem U Oksal A Kutluay L Uluoglu O Lin JC Unusually high frequency of a 69-bp deletion within the carboxy terminus of the LMP-1 oncogene of Epstein-Barr virus detected in Burkitt’s lymphoma of Turkish children Leukemia 1998 12 1796 1805 10.1038/sj.leu.2401203 9823956

[B26] Chen WG Chen YY Bacchi MM Bacchi CE Alvarenga M Weiss LM Genotyping of Epstein-Barr virus in Brazilian Burkitt’s lymphoma and reactive lymphoid tissue. Type A with a high prevalence of deletions within the latent membrane protein gene Am J Pathol 1996 148 17 23 8546204 PMC1861614

[B27] Bhatia K Raj A Guitierrez MI Judde JG Spangler G Venkatesh H Magrath IT Variation in the sequence of Epstein Barr virus nuclear antigen 1 in normal peripheral blood lymphocytes and in Burkitt’s lymphomas Oncogene 1996 13 177 181 8700544

[B28] Habeshaw G Yao QY Bell AI Morton D Rickinson AB Epstein-barr virus nuclear antigen 1 sequences in endemic and sporadic Burkitt’s lymphoma reflect virus strains prevalent in different geographic areas J Virol 1999 73 965 975 9882297 10.1128/jvi.73.2.965-975.1999 PMC103916

[B29] Chang CM Yu KJ Mbulaiteye SM Hildesheim A Bhatia K The extent of genetic diversity of Epstein-Barr virus and its geographic and disease patterns: a need for reappraisal Virus research 2009 143 209 221 10.1016/j.virusres.2009.07.005 19596032 PMC2731007

[B30] Rao CR Gutierrez MI Bhatia K Fend F Franklin J Appaji L Gallo G O’Conor G Lalitha N Magrath I Association of Burkitt’s lymphoma with the Epstein-Barr virus in two developing countries Leuk Lymphoma 2000 39 329 337 10.3109/10428190009065832 11342313

[B31] Lin H-J Cherng J-M Hung M-S Sayion Y Lin J-C Functional assays of HLA A2-restricted epitope variant of latent membrane protein 1 (LMP-1) of Epstein-Barr virus in nasopharyngeal carcinoma of Southern China and Taiwan J Biomed Sci 2005 12 925 936 10.1007/s11373-005-9017-y 16307312

[B32] Lin J-C Cherng J-M Lin H-J Tsang C-W Liu Y-X Lee SP Amino acid changes in functional domains of latent membrane protein 1 of Epstein-Barr virus in nasopharyngeal carcinoma of southern China and Taiwan: prevalence of an HLA A2-restricted “epitope-loss variant J Gen Virol 2004 85 Pt 7 2023 2034 15218188 10.1099/vir.0.19696-0

[B33] Pai S O’Sullivan B Abdul-Jabbar I Peng J Connoly G Khanna R Thomas R Nasopharyngeal carcinoma-associated Epstein-Barr virus-encoded oncogene latent membrane protein 1 potentiates regulatory T-cell function Immunol Cell Biol 2007 85 370 377 10.1038/sj.icb.7100046 17372611

[B34] Duraiswamy J Burrows JM Bharadwaj M Burrows SR Cooper L Pimtanothai N Khanna R Ex Vivo Analysis of T-Cell Responses to Epstein-Barr Virus-Encoded Oncogene Latent Membrane Protein 1 Reveals Highly Conserved Epitope Sequences in Virus Isolates from Diverse Geographic Regions J Virol 2003 77 7401 7410 10.1128/JVI.77.13.7401-7410.2003 12805439 PMC164808

[B35] Sandvej K Andresen BS Zhou X-G Gregersen N Hamilton-Dutoit S Analysis of the Epstein-Barr virus (EBV) latent membrane protein 1 (LMP-1) gene and promoter in Hodgkin’s disease isolates: selection against EBV variants with mutations in the LMP-1 promoter ATF-1/CREB-1 binding site Mol Pathol 2000 53 280 288 10.1136/mp.53.5.280 11091852 PMC1186981

[B36] Asito AS Piriou E Odada PS Fiore N Middeldorp JM Long C Dutta S Lanar DE Jura WG Ouma C Otieno JA Moormann AM Rochford R Elevated anti-Zta IgG levels and EBV viral load are associated with site of tumor presentation in endemic Burkitt’s lymphoma patients: a case control study Infect Agents Cancer 2010 5 13 10.1186/1750-9378-5-13 20667138 PMC2923120

[B37] Rainey JJ Mwanda WO Wairiumu P Moormann AM Wilson ML Rochford R Spatial distribution of Burkitt’s lymphoma in Kenya and association with malaria risk Tropical medicine & international health : TM & IH 2007 12 936 943 10.1111/j.1365-3156.2007.01875.x 17697088

[B38] Piriou E Asito AS Sumba PO Fiore N Middeldorp JM Moormann AM Ploutz-Snyder R Rochford R Early age at time of primary Epstein–Barr Virus infection results in poorly controlled viral infection in infants from Western Kenya: clues to the Etiology of Endemic Burkitt Lymphoma J Infect Dis 2012 205 906 913 10.1093/infdis/jir872 22301635 PMC3282570

[B39] Njie R Bell AI Jia H Croom-Carter D Chaganti S Hislop AD Whittle H Rickinson AB The effects of acute malaria on Epstein-Barr virus (EBV) load and EBV-specific T cell immunity in Gambian children J Infect Dis 2009 199 31 38 10.1086/594373 19032105

[B40] Mautner J Bornkamm GW The role of virus-specific CD4+ T cells in the control of Epstein-Barr virus infection Eur J Cell Biol 2012 91 31 35 10.1016/j.ejcb.2011.01.007 21458882

[B41] Zhang B Kracker S Yasuda T Casola S Vanneman M Hömig-Hölzel C Wang Z Derudder E Li S Chakraborty T Cotter SE Koyama S Currie T Freeman GJ Kutok JL Rodig SJ Dranoff G Rajewsky K Immune Surveillance and Therapy of Lymphomas Driven by Epstein-Barr Virus Protein LMP1 in a Mouse Model Cell 2012 148 739 751 10.1016/j.cell.2011.12.031 22341446 PMC3313622

[B42] Lemmermann NAW Kropp KA Seckert CK Grzimek NKA Reddehase MJ Reverse genetics modification of cytomegalovirus antigenicity and immunogenicity by CD8 T-cell epitope deletion and insertion J Biomed Biotechnol 2011 2011 812742 21253509 10.1155/2011/812742 PMC3021883

[B43] Matsumura M Fremont DH Peterson PA Wilson IA Emerging principles for the recognition of peptide antigens by MHC class I molecules Science 1992 257 927 934 10.1126/science.1323878 1323878

[B44] Cao K Moormann AM Lyke KE Masaberg C Sumba OP Doumbo OK Koech D Lancaster A Nelson M Meyer D Single R Hartzman RJ Plowe CV Kazura J Mann DL Sztein MB Thomson G Fernández-Viña MA Differentiation between African populations is evidenced by the diversity of alleles and haplotypes of HLA class I loci Tissue Antigens 2004 63 293 325 10.1111/j.0001-2815.2004.00192.x 15009803

[B45] Rainey JJ Omenah D Sumba PO Moormann AM Rochford R Wilson ML Spatial clustering of endemic Burkitt’s lymphoma in high-risk regions of Kenya International journal of cancerJournal international du cancer 2007 120 121 127 10.1002/ijc.22179 17019706

[B46] Moormann AM Chelimo K Sumba OP Lutzke ML Ploutz-Snyder R Newton D Kazura J Rochford R Exposure to holoendemic malaria results in elevated Epstein-Barr virus loads in children J Infect Dis 2005 191 1233 1238 10.1086/428910 15776368

